# Fellow-Workers and the Roll of Honour

**Published:** 1915-03-27

**Authors:** 


### ROUND THE WORLD.

[The Editor invites Early Information and Contributions Marked "Fellow-Workers."]

### Fellow-Workers and the Roll of Honour.

The Royal Army Medical Corps lost an experienced officer by the death of Major Francis Graham Richards, who fell in the recent severe fighting in France. A "Bart.'s" man, he qualified M.R.C.S., L.R.C.P. in 1899, and soon after entered the Army, where he quickly had an opportunity of witnessing active service, being present in several engagements, including the relief of Ladysmith and the Spion Kop affair. For his work during the Boer War Major Richards gained the Queen's medal (four clasps) and the King's medal (two clasps). He was only forty-one years old at the time of his death.

Lieutenant Gerald Quin Lennane, R.A.M.C. (T.F.), an officer of the 2nd London Sanitary Company, is the medical officer of health for Battersea, in which district he has been for some time a prominent figure. Qualifying in Ireland, and becoming an F.R.C.S.I., D.P.H., he came to London and held the posts of honorary casualty officer to the Queen's Jubilee Hospital, clinical assistant to the Royal Ophthalmic Hospital, and clinical assistant to the Chelsea Hospital for Women. Then taking up public health work he obtained the important public health appointment which he has maintained up to the present time.

Major Percy Samuel Lelean, R.A.M.C., who has been lecturing on "The Health of the Army in the Field" at the Millbank college, qualified from St. Mary's by taking the conjoint diploma in 1895, and subsequently became an F.R.C.S. Eng. and a D.P.H. In early years he was assistant demonstrator in physiology and resident casualty officer at St. Mary's Hospital, and house surgeon to the North Lonsdale Hospital in Furness. Major Lelean has had previous experience of military service as civil surgeon to the South African Field Force. Entering the Royal Army Medical Corps he gave a good deal of attention to public health work, and has lately been fulfilling the duties of sanitary officer to the Eastern Command.

Dr. D. F. Riddell, who is in charge of the small hospital for refugees in Sheffield Street, has lately been assistant medical officer to the Brook Fever Hospital. Graduating at Glasgow University in both arts and medicine in 1903 he subsequently became a D.P.H. Cantab., and has held the appointments of resident physician and resident surgeon to the Glasgow Royal Infirmary and assistant physician to the Glasgow Fever Hospital at Ruchill. The Kingsway Refugee Hospital is directed by the Metropolitan Asylums Board, under which Dr. Riddell has been serving at Shooter's Hill. In this new work he is assisted by a Belgian doctor, Dr. G. De Laey.

Serbia seems likely to deal hardly with those who have gone to assist her sick and wounded. It is to be regretted that Miss Margaret Ryle, who had been working for some time with the Russian Red Cross, has lost her life through an accident there after accomplishing excellent work on the trains carrying wounded from the Russian Front to the base hospitals. Her father, Dr. R. J. Ryle, is a Brighton practitioner who qualified from Oxford and Guy's Hospital in 1883, subsequently taking his M.D. degree. He holds the post of honorary surgeon to the Sussex Eye Hospital, and was formerly house surgeon at "Guy's."

The late Mr. W. Graham Moore Anderson, who was senior medical officer on the *Clan Macnaughton,* which went down in the North Sea during a gale, was a Trinity College, Dublin, man, and graduated M.B., B.Ch. in 1903. A son of Sir Robert Anderson, he joined the naval medical service soon after qualification, and was promoted to the rank of staff surgeon eight years after. He had only been with his ship a few weeks before the catastrophe in which he lost his life occurred.

The commandant of the Lady Hardinge Hospital for Indians at Brockenhurst, Lieutenant-Colonel Francis Frederick Perry, C.I.E., has had a distinguished connection with the Indian Medical Service, from which he retired some little time ago. A student of University College Hospital, he qualified in 1876, and became an F.R.C.S. Eng. in 1890. His academic successes include the Atkinson-Morley scholarship at his hospital, the Herbert scholarship and the Parkes and Martin medals at Netley, whilst in the course of his career he held the important posts of professor of surgery and ophthalmic surgery at Lahore Medical College and principal surgeon to the Mayo Hospital there. Before entering the I.M.S. he was house physician and house surgeon at University College Hospital, whilst at Westminster Hospital he held the posts of demonstrator in anatomy and surgical tutor.

During the recent disturbance at Singapore three members of the Colonial Medical Service lost their lives—namely, Dr. A. F. Legg, Dr. P. N. Gerrard, and Dr. E. D. Whittle.

Of these unfortunate officers, Dr. Percy Netterville Gerrard was senior. A Dublin man, he took the M.B. B.Ch. there in 1895, and three years later graduated M.D. Then he added to his clinical experience by serving as resident surgeon at the Royal City of Dublin Hospital, after which he entered the Colonial service as a medical officer of the Malay States, where he worked at Kuala Kangsar, Selangor, and Klang. A man who was keen on his work, Dr. Gerrard closely studied the problems of medicine in hot countries, and held the diplomas in tropical medicine both of the London and Cambridge Schools; in addition, he was the author of works on beri-beri, and "The Hygienic Management of Labour in the Tropics," among other publications. He also compiled a useful "Malay-English Vocabulary."

Dr. Edward Denis Whittle studied medicine at University College Hospital, and qualified M.R.C.S., L.R.C.P. in 1906. Subsequently he held the appointments of house surgeon and casualty medical officer at University College Hospital, and house surgeon to the Whitehaven and West Cumberland Infirmary. Then, going to the Malay States as an officer of the Colonial Medical Service, he eventually was given charge of Tan Tock Seng's Hospital at Singapore, and at the time of his death he was surgeon to the Singapore hospitals. His father, the late Dr. Whittle, used to practise in Brighton.

Dr. Angus Forsyth Legge was a new-comer in Singapore. He qualified M.B., Ch.B. of Aberdeen in 1912.

